# Are the effects of blood pressure lowering treatment diminishing?: meta-regression analyses

**DOI:** 10.1186/s40885-018-0101-9

**Published:** 2018-11-15

**Authors:** Yoichi Sekizawa, Yoko Konishi, Moriyo Kimura

**Affiliations:** 1 Research Institute of Economy, Trade and Industry, 1-3-1 Kasumigaseki Chiyoda-ku, Tokyo, 100-8901 Japan; 2 The Public Health Institute, 3-3-23-104, Sendagaya Shibuya-ku, Tokyo, 151-0051 Japan

**Keywords:** Blood pressure lowering treatment, Temporal trend, Diminishing effect, All-cause mortality, Stroke, Meta-regression

## Abstract

**Background:**

Previous studies have suggested that the effects of medical interventions tend to diminish over time. We investigated whether the effects of blood pressure lowering treatment on all-cause mortality and stroke have diminished over time.

**Methods:**

We conducted meta-regression analyses. We extracted the target trials from two recently published comprehensive systematic reviews and meta-analyses. Adopted variables were relative risk (RR) of all-cause mortality and stroke, trial start year, mean age, sample size, baseline systolic blood pressure (SBP), difference in attained SBP reduction between intervention groups and control groups (SBP difference), and regional dummies. We implemented single meta-regressions, in which the dependent variable was the log of RR and the explanatory variable was each of other adopted variables. We also conducted multiple meta-regressions, in which the dependent variable was the log of RR and explanatory variables were all of other adopted variables. Our variable of greatest interest was trial start year.

**Results:**

The included reviews assessed 85 trials with a total of 343,126 participants. Although trial start year was positively associated with the log of RR in the results of single meta-regressions, it lost significance in multiple meta-regressions for both all-cause mortality and stroke.

**Conclusions:**

The effects of blood pressure lowering treatment on all-cause mortality and stroke have not diminished over time.

**Electronic supplementary material:**

The online version of this article (10.1186/s40885-018-0101-9) contains supplementary material, which is available to authorized users.

## Background

Previous studies suggest that the effects of medical interventions tend to diminish over time [1–7]. For example, recent analyses using meta-regression of the relevant trials suggested that the effects of antidepressants and cognitive behavioral therapy as treatments for depression diminished over time [8–11]. Nevertheless, whether this diminishing phenomenon can be observed in other medical fields seems to be underexplored.

Given this, we investigated whether the effects of blood pressure lowering treatments on all-cause mortality and stroke have diminished over time. Numerous clinical trials and meta-analyses indicated that blood pressure lowering treatments lessen serious cardiovascular events and all-cause mortality [12, 13]. To our knowledge, however, it has not been sufficiently explored in previous studies whether the effect of blood pressure lowering treatment has changed over time.

Therefore, the objective of this study was to examine whether the effects of blood pressure lowering treatment on all-cause mortality and stroke have diminished over time.

## Methods

### Ideas for inclusion of trials

Although there are several systematic reviews and meta-analyses regarding the effects of blood pressure lowering treatment on cardiovascular diseases and mortality, the most recent and comprehensive we are aware of are Ettehad et al. [13], which was published in 2016, and Brunström and Carlberg [14], which was published in 2018.

The Ettehad paper included a whole range of randomized controlled trials on blood pressure lowering treatment. The authors searched MEDLINE from January 1, 1966 till July 7, 2015. They also searched the medical literature and identified trials till November 9, 2015. Their search strategy was compatible to that of Law et al. [12] and used search terms related to hypertension, blood pressure, major classes of blood pressure lowering drugs (for example, diuretics), and the specific names of blood pressure lowering drugs listed in the British National Formulary. The studies Ettehad et al. included had a minimum of 1000 patient-years of follow-up in each trial group. The paper selected trials which compared active drug treatment groups with control groups, which mostly used placebo. The paper also selected trials which compared groups with different blood pressure targets. Although the paper did not exclude trials based on the existence of comorbidities at baseline, they excluded trials in patients with heart failure or left ventricular dysfunction.

The Brunström and Carlberg paper identified previous systematic reviews on the effect of blood pressure lowering from PubMed, the Cochrane Database of Systematic Reviews, and the Database of Abstracts of Reviews of Effect. They identified trials by scrutinizing reference lists from relevant reviews. They additionally searched PubMed and Cochrane Central Register for Controlled Trials in order to find trials after November 1, 2015 up till February 2017. The Brunström and Carlberg paper included mostly the same type of trials as the Ettehad paper, except for the following points. Unlike the Ettehad paper, the Brunström and Carlberg paper excluded trials in the acute phase after myocardial infarction from meta-analysis. They also excluded trials they deemed to be at high risk of bias. Unlike the Ettehad paper, which required a minimum of 1000 patient-years of follow-up for each trial group, the Brunström and Carlberg paper required a minimum of 1000 patient-years of follow-up for the whole study sample. We constructed our dataset for meta-analysis and meta-regression by adopting the trials included in these two papers. We used the names of included trials as used in the Ettehad paper and/or the Brunström and Carlberg paper.

### Data extraction

One of the authors (YS) extracted the following data from each trial: trial start year, sample size of trial (number of total participants), number of events (all-cause mortality and stroke), mean age at baseline, mean systolic blood pressure (SBP) at baseline, difference in SBP reduction between the two groups, SBP reduction from the baseline in each group, attained SBP in each group, and the region in which each trial was carried out.

As defined in both the Ettehad paper and the Brunström and Carlberg paper, stroke included fatal and non-fatal stroke and excluded transient ischemic attack (TIA). In addition to data on stroke as a whole, we aimed to extract data on its subtypes (e.g., cerebral infarction, cerebral hemorrhage, and subarachnoid hemorrhage) from each trial and, where possible, performed analyses based on the subtypes. In the process of data extraction, we found that several papers covered only fatal stroke or non-fatal stroke. For these papers, we recorded the cases shown in each paper. We also found that several papers included TIA in the number of cases, and exclusion of TIA was not possible. In these cases, we extracted the number of cases including TIA.

As the Ettehad paper showed the number of events (all-cause mortality and stroke) in its appendix, we compared our extraction results with those of the Ettehad paper. When there were differences, we explored the causes of the differences. This exploration revealed that some data were different from those in the Ettehad paper. Regarding all-cause mortality, there were three differences compared with the Ettehad paper (Supp. Figure 11 [13]). In PREVEND IT [15], we calculated the number of non-cardiovascular deaths from the indicated ratio and added them to the cardiovascular death number. In BBB [16], we could not extract all-cause mortality data because it was not indicated in the paper. In EWPHE [17], we used the number from the intention-to-treat analysis. Regarding stroke, there were five differences compared with the Ettehad paper (Supp. Figure 8 [13]). In PHARAO [18], we added hemorrhage. In Oslo [19], we added fatal subarachnoidal hemorrhage. In JATOS [20], we subtracted the number of TIA. In EWPHE [17], we used the numbers from the intention-to-treat analysis. In the Hunan Province study [21], we used the data indicated in the original publication.

Regarding the variable to identify when each trial was carried out, we used the data of trial start year. To extract the trial start year, we first checked the papers for each trial. When we could not find the trial start year, we checked ClinicalTrials.gov. When these did not work, we emailed the corresponding or first author of the original papers.

Differences in the attained SBP reduction between intervention groups and control groups (hereinafter, “SBP difference”) were determined in the following way. We retrieved three patterns of figures for SBP difference: (1) difference in SBP reduction between the two groups, (2) SBP reduction from the baseline in each group, and (3) attained SBP in each group. We prioritized (1) when reported. If (1) was not reported, we calculated the difference from (2). If neither (1) nor (2) were reported, we calculated the difference from (3). We used mean figures of the whole measurements when reported. When plural measurements were reported and mean figures were not reported, we calculated the mean of plural measurements. When only graphs of SBP transition were shown, we calculated the SBP levels using an online tool [22]. Regarding the Lewis study [23], SBP data were extracted from Black et al. [24]. In BBB [16], the number in each group was not mentioned, so we split the total amount (*N* = 2127) and assumed both groups included 1064 participants.

The regions in which trials were carried out were categorized as follows: Asia, Europe, North America, Oceania, South America, and mixed regions. Trials carried out in two or more regions were included in the mixed region category. Turkey and Israel were included in Europe.

### Statistical analysis

The statistical analyses in this study were carried out in the following two steps. First, we conducted single meta-regressions, in which the dependent variable was the log RR of all-cause mortality or stroke and the explanatory variable was each of the extracted variables, namely trial start year, sample size, age, baseline SBP, SBP difference, and region (Model 1M for all-cause mortality and Model 1S for stroke). RR was calculated by dividing the risk of outcome events of intervention groups by the risk of outcome events of control groups. Risk of outcome events of each group was calculated by dividing the number of participants experiencing outcome events by the number of participants. With regard to the variable of region, there were several variables for regional dummies in the model, but we included it in the table of single meta-regression because they all belonged to the regional category.

Second, we conducted multiple meta-regressions, in which the dependent variable was the log RR and the explanatory variables were all of the abovementioned variables (Model 2M for all-cause mortality and Model 2S for stroke). We judged whether the effects of blood pressure lowering treatment on all-cause mortality and stroke have diminished over time from Model 2M and Model 2S. Diminishing was confirmed only when the variable of trial start year was significant at the 5% level.

When there was no event in either group in a trial, we added 0.5 to the number of events and the number of non-events in both groups before performing the meta-regressions. All analyses were performed using STATA 15 (Stata Corp., College Station, TX, USA). For meta-regressions, we used the “metareg” command [25], which estimates the random-effects model via the restricted maximum likelihood method.

For trials with three or more arms, we followed the classification performed in the Ettehad paper or the Brunström and Carlberg paper. The two papers defined the control group in the ONTARGET study [26] differently; here, we followed the Brunström and Carlberg paper’s classification.

## Results

### Number of trials and participants

The list of all trials in our study is shown in Additional file 1: Table S1. Data for trial start year, sample size, mean age, mean baseline SBP, mean SBP difference of each trial, and the region in which each trial was carried out are also shown in Additional file 1: Table S1. Outcome events and the number of participants of intervention groups and control groups are shown in Additional file 2: Table S2 for all-cause mortality and in Additional file 3: Table S3 for stroke.

A total of 85 trials were selected, representing 343,126 participants. Among these, there were 82 trials for all-cause mortality and 74 trials for stroke. Summary statistics of the trials are shown in Table 1.

**Table 1:** Summary statistics of the included trials

	*K*	mean (sd)	minimum	maximum
All trials				
Trial start year (years)	82	1993.0 (10.3)	1964	2010
Sample size (number of participants)	85	4036.8 (4919.6)	194	25,620
Age (years)	84	61.0 (10.0)	29.7	83.8
Baseline SBP (mmHg)	84	148.9 (18.5)	112.0	196.4
SBP difference (mmHg)	81	7.3 (6.2)	−0.1	31.4
Region	11 in Asia, 27 in Europe, 16 in North America, 2 in Oceania, 1 in South America, 28 in mixed regions			
Trials on all-cause mortality				
Trial start year (years)	79	1993.0 (10.4)	1964	2010
Sample size (number of participants)	82	4119.0 (4989.8)	194	25,620
Age (years)	81	61.0 (10.1)	29.7	83.8
Baseline SBP (mmHg)	81	148.6 (18.8)	112.0	196.4
SBP difference (mmHg)	78	7.3 (6.2)	−0.1	31.4
Region (mmHg)	9 in Asia, 26 in Europe, 16 in North America, 2 in Oceania, 1 in South America, 28 in mixed regions			
Trials on stroke				
Trial start year (years)	71	1992.8 (10.3)	1964	2010
Sample size (number of participants)	74	4459.0 (5133.6)	281	25,620
Age (years)	73	62.6 (8.7)	29.7	83.8
Baseline SBP (mmHg)	74	150.9 (18.1)	112.0	196.4
SBP difference (mmHg)	71	7.9 (6.3)	−0.1	31.4
Region	11 in Asia, 22 in Europe, 15 in North America, 2 in Oceania, 24 in mixed regions			

*K* = number of trials; sd = standard deviation

### Results of meta-regressions for all-cause mortality

In Table 2, we reported the results of meta-regressions for all-cause mortality. The dependent variable was log RR of all-cause mortality. With the increase in RR, the effect of blood pressure lowering treatment is interpreted to decrease. In the table, exponentiated coefficients (exp(b)) instead of coefficients per se are reported. Exp(b) is the exponential of the unstandardized coefficient and shows a proportional change in RR per unit change in each explanatory variable. This makes more straightforward interpretation possible by denoting the multiplicative effects upon RR. If exp(b) is greater than 1, when the explanatory variable rises by one unit, RR increases (effect of blood pressure lowering treatment on all-cause mortality diminishes) by the ratio of exp(b) minus one. If exp(b) is smaller than 1, when the explanatory variable rises by one unit, RR falls (effect of blood pressure lowering treatment on all-cause mortality increases) by the ratio of one minus exp(b).

**Table 2:** Meta-regression analyses (Dependent variable: natural log of relative risk for all-cause mortality)

	Model 1M (single)			Model 2M (multiple)		
Variable	exp(b)	95% CI	*p-*value	exp(b)	95% CI	*p-*value
Trial start year (per 10 years)	1.05	1.02–1.09	0.004	1.05	0.99–1.10	0.078
	Adjusted R^2^ = 34.8%					
Age (per 10 years)	1.01	0.96–1.07	0.633	0.97	0.91–1.03	0.311
	Adjusted R^2^ = −1.8%					
Sample size (per 1000 participants)	1.01	1.00–1.01	0.012	1.00	1.00–1.01	0.188
	Adjusted R^2^ = 32.7%					
Baseline SBP (per 10 mmHg)	0.99	0.97–1.01	0.414	1.02	0.99–1.05	0.201
	Adjusted R^2^ = −2.3%					
SBP difference (per 10 mmHg)	0.93	0.87–0.99	0.033	1.00	0.90–1.11	0.990
	Adjusted R^2^ = 28.3%					
Region (per category)						
Mixed region	1 (ref)			1 (ref)		
North America	0.85	0.77–0.93	0.001	0.89	0.78–1.00	0.055
Europe	0.93	0.85–1.00	0.062	0.95	0.85–1.06	0.341
Asia	0.80	0.70–0.91	0.001	0.84	0.73–0.97	0.020
Oceania	0.69	0.46–1.04	0.077			
South Africa	0.98	0.06–16.9	0.988	0.94	0.05–16.3	0.968
	Adjusted R^2^ = 61.9%					
Adjusted R^2^				68.9%		
*K*				74		

*exp(b)* the exponentiation of the b coefficient; *ref.* reference; *CI* confidence interval; *K* number of trials

The results of single meta-regressions (Model 1M in Table 2), in which other explanatory variables were not adjusted for, are as follows. Regarding the trial start year, which was the explanatory variable of greatest interest, RR rose with the increase in trial start year; for every 10 years added to the trial start year, RR of all-cause mortality increased significantly by 5% (exp(b) = 1.05; 95% CI 1.02, 1.09; *p* = 0.004) in the case of no adjustment for other explanatory variables. For other explanatory variables, RR significantly grew with the rise in sample size and decreased with the enlarged SBP difference. RR was significantly lower in trials carried out in North America and Asia than in mixed region trials.

According to multiple meta-regression that included all of the explanatory variables, the association between trial start year and log RR of all-cause mortality lost significance (exp(b) = 1.05; 95% CI 0.99, 1.10; *p* = 0.078) after adjusting for other explanatory variables. Similarly, associations of log RR of all-cause mortality with sample size, SBP difference, and regional dummies for North America lost significance after adjusting for other explanatory variables. Trials in Asia still had a significantly lower log RR than mixed region trials after adjusting for other explanatory variables; RR of all-cause mortality of trials in Asia was 16% lower than mixed region trials (exp(b) = 0.84; 95% CI 0.73, 0.97; *p* = 0.020) after adjusting for other explanatory variables (Model 2M in Table 2).

As the trial start year variable was not significant at the 5% level in multiple meta-regression, we concluded that the effects of blood pressure lowering treatment on all-cause mortality have not diminished over time.

### Results of meta-regressions for stroke

In Table 3, we reported the results of meta-regressions for stroke. The dependent variable was log RR of stroke. Results of single meta-regressions (Model 1S in Table 3), in which other explanatory variables were not adjusted for, are as follows. Regarding the trial start year which is the explanatory variable of greatest interest, RR rose with the increase in trial start year; for every 10 years added to the trial start year, RR of stroke grew significantly by 12% (exp(b) = 1.12; 95% CI 1.06, 1.19; *p* <  0.001) in the case of no adjustment for other explanatory variables. For other explanatory variables, RR significantly climbed with the rise in sample size and fell with the growing baseline SBP and SBP difference. RR was significantly lower in trials carried out in North America, Europe, and Asia than in mixed region trials.

**Table 3:** Meta-regression analyses (Dependent variable: natural log of relative risk for stroke)

	Model 1S (single)			Model 2S (multiple)		
Variable	exp(b)	95% CI	*p-*value	exp(b)	95% CI	*p-*value
Trial start year (per 10 years)	1.12	1.06–1.19	< 0.001	1.07	0.97–1.19	0.154
	Adjusted R^2^ = 50.5%					
Age (per 10 years)	1.02	0.93–1.13	0.642	1.00	0.89–1.12	0.973
	Adjusted R^2^ = −1.5%					
Sample size (per 1000 participants)	1.01	1.00–1.02	0.005	1.00	0.99–1.01	0.509
	Adjusted R^2^ = 30.9%					
Baseline SBP (per 10 mmHg)	0.94	0.91–0.98	0.003	1.03	0.97–1.09	0.399
	Adjusted R^2^ = 28.4%					
SBP difference (per 10 mmHg)	0.78	0.71–0.86	< 0.001	0.81	0.69–0.94	0.008
	Adjusted R^2^ = 60.5%					
Region (per category)						
Mixed region	1 (ref)			1 (ref)		
North America	0.81	0.68–0.97	0.024	1.05	0.85–1.29	0.649
Europe	0.81	0.69–0.94	0.008	0.97	0.81–1.17	0.782
Asia	0.78	0.66–0.93	0.005	0.83	0.70–0.99	0.042
Oceania	0.89	0.46–1.70	0.713			
	Adjusted R^2^ = 40.2%					
Adjusted R^2^				79.8%		
*K*				67		

*exp(b)* the exponentiation of the b coefficient; *ref*. reference; *CI* confidence interval; *K* number of trials

According to multiple meta-regression that included all of the explanatory variables, the association between trial start year and log RR of stroke lost significance (exp(b) = 1.07; 95% CI 0.97, 1.19; *p* = 0.154) after adjusting for other explanatory variables. Similarly, associations of log RR of stroke with sample size, baseline SBP, and regional dummies for North America and Europe lost significance after adjusting for other explanatory variables. SBP difference still had a significant association with log RR; for every 10 mmHg increase in SBP difference, RR of stroke decreased significantly by 19% (exp(b) = 0.81; 95% CI 0.69, 0.94; *p* = 0.008) after adjusting for other explanatory variables. Trials in Asia still had a significantly lower log RR than mixed region trials after adjusting for other explanatory variables; RR of stroke for trials in Asia was 17% lower than mixed region trials (exp(b) = 0.83; 95% CI 0.70, 0.99; *p* = 0.042) after adjusting for other explanatory variables (Model 2S in Table 3).

As the trial start year variable was not significant at the 5% level in multiple meta-regression, we concluded that the effects of blood pressure lowering treatment on stroke have not diminished over time.

Regarding analyses by stroke subtypes, out of the 74 trials we covered, we found only five trials [20, 27–30] which indicated data based on ischemic type and hemorrhagic type. Hence, we discontinued our analyses of stroke subtypes.

## Discussion

In this study, we investigated whether the effects of blood pressure lowering treatment on all-cause mortality and stroke have diminished over time by performing meta-regressions in which log RRs of the outcome events were the dependent variable and trial start year was the explanatory variable of greatest interest. Although trial start year was positively associated with log RR in the results of single meta-regressions, it lost significance in multiple meta-regressions which adjusted for mean age, sample size, baseline SBP, SBP difference, and regional dummies for both all-cause mortality and stroke. We concluded that the effects of blood pressure lowering treatment on all-cause mortality and stroke have not diminished over time from the results of the multiple meta-regressions.

In this study, we used the data of trial start year instead of year of publication as the variable to identify when each trial was performed. One problem in using data of trial start year was that some studies do not report this variable in published papers. Although we did our best to identify trial start year by checking the ClinicalTrials.gov website and emailing the corresponding authors, we were still unable to identify the trial start year for three trials. This may have caused some bias and/or lessened statistical power.

In this study, SBP difference (difference in attained SBP reduction between intervention groups and control groups) was not associated with risk reduction of all-cause mortality in multiple meta-regression. Although Ettehad et al. [13] indicated that there was a significant association between the extent of SBP reduction and improved RR outcome in all-cause mortality from single meta-regression analysis, and we also found similar results in our single meta-regression, the results gained from single meta-regression were not supported by multiple meta-regression analysis as done in this study. By contrast, our results on stroke, which showed that SBP difference was associated with a lower RR of stroke in both single and multiple meta-regressions, support and strengthen the findings of Ettehad et al. [13] and another meta-regression analysis [31].

In this study, trials in Asia showed significantly lower RR compared with mixed region trials in both all-cause mortality and stroke. Of the 11 trials carried out in Asia, six were from China, four from Japan, and one from Hong Kong and Japan. The reasons why such higher effects are shown in Asia need to be explored in future studies.

There are several limitations to this study. First, we included the trials covered by two recent comprehensive systematic reviews. Additional trials not covered by the included systematic reviews may alter these findings. Second, the definition of stroke varied among trials. Variation in definition may have caused biases in our meta-regression on stroke. Third, since most trials included participants with comorbidities, the trials covered in this study might be heterogeneous. Fourth, we assumed a linear relationship between log RRs and trial start year. This assumption may be questioned, and the analysis could be improved by better reflecting the behavior of the data when constructing the model. Fifth, adjusted R^2^ for multiple regressions was not large enough (68.9% for all-cause mortality and 79.8% for stroke), suggesting the possibility of other important factors which would substantially change the results of this paper.

## Conclusions

The effects of blood pressure lowering treatment on all-cause mortality and stroke have not diminished over time.

## Additional files

Additional file 1: **Table S1.** Summary of Included Trials. (DOCX 31 kb)

Additional file 2: **Table S2.** Summary of Outcome Events on All-cause Mortality. (DOCX 28 kb)

Additional file 3: **Table S3.** Summary of Outcome Events on Stroke. (DOCX 28 kb)

## Abbreviations

CI: Confidence interval
RR: Relative risk
SBP difference: Difference in attained SBP reduction between intervention groups and control groups
SBP: Systolic blood pressure
TIA: Transient ischemic attack
