# Supplementary material for: Are the effects of blood pressure lowering treatment diminishing?: meta-regression analyses
Source: Clin Hypertens. 2018 Nov 15;24:16. doi: 10.1186/s40885-018-0101-9 (PMC6237040; doi:10.1186/s40885-018-0101-9)
Supplement: Supplementary file 1 — Table S1. Summary of Included Trials. (DOCX 31 kb) [file 40885_2018_101_MOESM1_ESM.docx]

| **Additional file 1**  Table S1. Summary of Included Trials | | | | | |  |
| --- | --- | --- | --- | --- | --- | --- |
| Trial Name | Trial Start Year | Mean Age | Sample Size | Mean Baseline SBP | Mean　SBP difference | Region |
| AASK [1, 2] | 1995 | 54.6 | 1094 | 150.5 | 13.0 | North America |
| ABCD-H [3, 4] | 1991 | 57.9 | 470 | 155.0 | 6.0 | North America |
| ABCD-N [5] | 1991 | 59.1 | 480 | 136.4 | 9.0 | North America |
| ACCORD [6] | 2001 | 62.2 | 4733 | 139.2 | 14.2 | North America |
| ACTION [7] | 1996 | 63.4 | 7665 | 137.5 | 5.5 | mixed region |
| ACTIVE I [8] | 2003 | 69.5 | 9016 | 138.3 | 2.9 | mixed region |
| ADVANCE [9] | 2001 | 66.0 | 11140 | 145.0 | 5.6 | mixed region |
| AIPRI [10] | 1989 | 51.0 | 583 | 143.0 | 9.6 | Europe |
| ALTITUDE [11] | 2007 | 64.5 | 8561 | 137.3 | 1.3 | mixed region |
| ANBPS [12] | 1973 | 50.4 | 3427 | 157.4 | N.A. | Oceania |
| BBB [13] | 1987 | 59.8 | 2127 | 155.0 | 11.0 | Europe |
| BCAPS [14] | 1991 | 61.8 | 793 | 138.9 | 1.3 | Europe |
| BENEDICT [15] | 1997 | 62.3 | 1204 | 150.8 | 2.3 | Europe |
| BENEDICT-B [16] | 1998 | 62.4 | 281 | 149.5 | -0.1 | Europe |
| BHAT [17, 18] | 1978 | 54.8 | 3837 | 112.0 | 3.0 | North America |
| CAMELOT [19] | 1999 | 57.7 | 1991 | 129.1 | 5.6 | mixed region |
| Cardio-Sis [20] | 2005 | 67.0 | 1111 | 163.3 | 3.8 | Europe |
| DAVIT 2 [21] | 1985 | 59.7 | 1775 | 120.0 | 5.0 | Europe |
| DEMAND [22] | 2002 | 60.8 | 380 | 147.5 | 2.0 | Europe |
| DIABHYCAR [23] | 1995 | 65.1 | 4912 | 145.4 | 2.0 | mixed region |
| DIRECT PREVENT 1 [24] | 2001 | 29.7 | 1421 | 116.0 | 2.6 | mixed region |
| DIRECT-PROTECT 1 [24] | 2001 | 31.7 | 1905 | 117.0 | 3.6 | mixed region |
| DIRECT-PROTECT 2 [25, 26] | 2001 | 57.0 | 1905 | 132.9 | 3.4 | mixed region |
| DREAM [27] | 2001 | 54.7 | 5269 | 136.0 | 4.3 | mixed region |
| Dutch TIA [28] | 1986 | N.A. | 1473 | 157.5 | 5.8 | Europe |
| EUROPA [29] | 1997 | 60.0 | 12218 | 137.0 | 5.0 | Europe |
| EWPHE [30] | 1978 | 72.0 | 840 | 182.5 | 21.0 | Europe |
| FEVER [31] | 1999 | 61.5 | 9711 | 154.3 | 4.2 | Asia |
| Fogari-02 [32] | N.A. | 62.5 | 309 | 160.3 | 10.1 | Europe |
| HDFP [33] | 1973 | 50.8 | 10940 | 159.0 | 11.0 | North America |
| HEP [34] | 1978 | 68.8 | 884 | 196.4 | 18.0 | Europe |
| HOMED-BP [35] | 2001 | 59.6 | 3518 | 154.2 | 1.1 | Asia |
| HOPE [36] | 1993 | 66.0 | 9297 | 139.0 | 3.3 | mixed region |
| HOPE-3 [37] | 2007 | 65.7 | 12705 | 138.1 | 6.0 | mixed region |
| HOT [38] | 1992 | 61.5 | 18790 | 169.7 | 2.7 | mixed region |
| HSCS [39] | 1966 | 59.0 | 452 | 167.0 | 25.3 | North America |
| Hunan Province [40] | 1990 | 51.8 | 2080 | 160.5 | 8.2 | Asia |
| HYVET [41] | 2000 | 83.6 | 3845 | 173.0 | 15.0 | mixed region |
| HYVET pilot [42] | 1994 | 83.8 | 1283 | 181.5 | 23.0 | Europe |
| IDNT [43, 44] | 1996 | 58.9 | 1715 | 159.0 | 3.5 | mixed region |
| IMAGINE [45] | 1999 | 61.0 | 2553 | 121.5 | 3.9 | mixed region |
| IPPPSH [46] | 1977 | 52.2 | 6357 | 173.0 | 3.8 | mixed region |
| IRMA-2 [47] | 1997 | 58.0 | 590 | 153.0 | 2.0 | mixed region |
| JATOS [48] | 2001 | 73.6 | 4418 | 171.6 | 9.7 | Asia |
| Lewis-93 [49] | 1987 | 34.5 | 409 | 138.5 | 1.3 | North America |
| MACB [50] | 1988 | 64.0 | 967 | 120.0 | N.A. | Europe |
| MRC-1 [51] | 1977 | 52.0 | 17354 | 161.4 | 11.3 | Europe |
| MRC-2 [52] | 1982 | 70.3 | 4396 | 184.7 | 14.6 | Europe |
| MultiCentre Int [53] | 1972 | 55.0 | 3038 | 123.0 | 4.0 | Europe |
| NAVIGATOR [54] | 2002 | 63.8 | 9306 | 139.7 | 2.8 | mixed region |
| ONTARGET [55] | 2001 | 66.4 | 25620 | 141.8 | 2.0 | mixed region |
| ORIENT [56] | 2003 | 59.2 | 566 | 141.2 | 2.8 | Asia |
| OSCAR [57] | 2005 | 73.6 | 1164 | 157.7 | 2.4 | Asia |
| Oslo [58] | 1972 | 45.3 | 785 | 155.8 | 17.0 | Europe |
| PART-2 [59] | N.A. | 60.5 | 617 | 133.0 | 6.0 | Oceania |
| PATS [60] | 1989 | 60.2 | 5665 | 153.8 | 6.4 | Asia |
| PEACE [61] | 1996 | 64.0 | 8290 | 133.5 | 3.0 | mixed region |
| PHARAO [62] | 2000 | 62.2 | 1008 | 134.4 | 2.8 | Europe |
| PREVEND IT [63] | 1997 | 51.0 | 864 | 130.0 | 5.6 | Europe |
| PREVENT [64] | 1992 | 56.9 | 825 | 129.4 | 8.0 | North America |
| PREVER [65] | 2010 | 50.0 | 730 | 127.3 | 2.2 | South America |
| PRoFESS [66] | 2003 | 66.2 | 20332 | 144.2 | 3.8 | mixed region |
| PROGRESS [67] | 1995 | 64.0 | 6105 | 147.0 | 9.0 | mixed region |
| QUIET [68] | 1991 | 58.0 | 1750 | 123.0 | N.A. | mixed region |
| RASS [69] | 1997 | 29.7 | 285 | 119.7 | 3.0 | North America |
| RAVID-98 [70] | 1990 | 55.0 | 194 | N.A. | N.A. | Europe |
| RENAAL [71] | 1996 | 60.0 | 1513 | 152.5 | 2.3 | mixed region |
| ROADMAP [72] | 2004 | 57.7 | 4447 | 136.5 | 3.3 | Europe |
| SCAT [73] | 1991 | 61.0 | 460 | 130.0 | 3.9 | North America |
| SCOPE [74] | 1997 | 76.4 | 4937 | 166.2 | 3.2 | mixed region |
| SHEP [75, 76] | 1985 | 71.5 | 4736 | 170.3 | 12.4 | North America |
| SHEP pilot [77] | 1981 | 72.1 | 551 | 172.0 | 15.5 | North America |
| SPRINT [78] | 2010 | 67.9 | 9361 | 139.7 | 13.1 | North America |
| SPS3 [79] | 2003 | 63.0 | 3020 | 143.0 | 11.0 | mixed region |
| STONE [80] | 1987 | 66.4 | 1632 | 169.0 | 9.3 | Asia |
| STOP [81] | 1985 | 75.6 | 1627 | 195.0 | 19.5 | Europe |
| Syst-China [82, 83] | 1988 | 66.5 | 2394 | 170.5 | 8.6 | Asia |
| Syst-Eur [84] | 1989 | 70.3 | 4695 | 173.8 | 10.4 | Europe |
| TEST [85] | 1988 | 70.4 | 720 | 161.0 | 4.0 | Europe |
| TRANSCEND [86] | 2001 | 66.9 | 5926 | 141.0 | 4.0 | mixed region |
| UKPDS 38 [87] | 1987 | 56.4 | 1148 | 159.3 | 10.0 | Europe |
| VA NEPHRON-D [88] | 2008 | 64.6 | 1448 | 137.0 | 1.5 | North America |
| VA-2 [89] | 1964 | 51.3 | 380 | 163.6 | 31.4 | North America |
| VALISH [90] | 2004 | 76.1 | 3079 | 169.5 | 5.6 | Asia |
| Wei-13 [91] | N.A. | 76.6 | 724 | 159.5 | 14.0 | Asia |

**References**

1. Wright, Jr JT, Bakris G, Greene T, et al. Effect of blood pressure lowering and antihypertensive drug class on progression of hypertensive kidney disease: Results from the aask trial. JAMA. 2002;288:2421-31. doi: 10.1001/jama.288.19.2421.

2. Norris K, Bourgoigne J, Gassman J, Hebert L, Middleton J, Phillips RA, et al. Cardiovascular Outcomes in the African American Study of Kidney Disease and Hypertension (AASK) Trial. Am J Kidney Dis. 2006;48:739-51. doi: 10.1053/j.ajkd.2006.08.004.

3. Estacio RO, Jeffers BW, Hiatt WR, Biggerstaff SL, Gifford N, Schrier RW. The effect of nisoldipine as compared with enalapril on cardiovascular outcomes in patients with non-insulin-dependent diabetes and hypertension. N Engl J Med. 1998;338:645-52. doi: 10.1056/nejm199803053381003.

4. Estacio RO, Jeffers BW, Gifford N, Schrier RW. Effect of blood pressure control on diabetic microvascular complications in patients with hypertension and type 2 diabetes. Diabetes Care. 2000;23 Suppl 2:B54-64.

5. Schrier RW, Estacio RO, Esler A, Mehler P. Effects of aggressive blood pressure control in normotensive type 2 diabetic patients on albuminuria, retinopathy and strokes. Kidney Int. 2002;61:1086-97. doi: 10.1046/j.1523-1755.2002.00213.x.

6. Cushman WC, Evans GW, Byington RP, Goff DC, Jr., Grimm RH, Jr., Cutler JA, et al. Effects of intensive blood-pressure control in type 2 diabetes mellitus. N Engl J Med. 2010;362:1575-85. doi: 10.1056/NEJMoa1001286.

7. Poole-Wilson PA, Lubsen J, Kirwan B-A, van Dalen FJ, Wagener G, Danchin N, et al. Effect of long-acting nifedipine on mortality and cardiovascular morbidity in patients with stable angina requiring treatment (ACTION trial): randomised controlled trial. The Lancet. 2004;364:849-57. doi: 10.1016/S0140-6736(04)16980-8.

8. Yusuf S, Healey JS, Pogue J, Chrolavicius S, Flather M, Hart RG, et al. Irbesartan in patients with atrial fibrillation. N Engl J Med. 2011;364:928-38. doi: 10.1056/NEJMoa1008816.

9. Patel A. Effects of a fixed combination of perindopril and indapamide on macrovascular and microvascular outcomes in patients with type 2 diabetes mellitus (the ADVANCE trial): a randomised controlled trial. The Lancet. 2007;370:829-40. doi: 10.1016/S0140-6736(07)61303-8.

10. Maschio G, Alberti D, Janin G, Locatelli F, Mann JF, Motolese M, et al. Effect of the angiotensin-converting-enzyme inhibitor benazepril on the progression of chronic renal insufficiency. The Angiotensin-Converting-Enzyme Inhibition in Progressive Renal Insufficiency Study Group. N Engl J Med. 1996;334:939-45. doi: 10.1056/nejm199604113341502.

11. Parving HH, Brenner BM, McMurray JJ, de Zeeuw D, Haffner SM, Solomon SD, et al. Cardiorenal end points in a trial of aliskiren for type 2 diabetes. N Engl J Med. 2012;367:2204-13. doi: 10.1056/NEJMoa1208799.

12. The Australian therapeutic trial in mild hypertension. Report by the Management Committee. Lancet. 1980;1:1261-7.

13. Hansson L. The BBB Study: The Effect of Intensified Antihypertensive Treatment on the Level of Blood Pressure, Side-Effects, Morbidity and Mortality in “Well-Treated” Hypertensive Patients. Blood Press. 1994;3:248-54. doi: 10.3109/08037059409102265.

14. Hedblad B, Wikstrand J, Janzon L, Wedel H, Berglund G. Low-Dose Metoprolol CR/XL and Fluvastatin Slow Progression of Carotid Intima-Media Thickness. Main Results From the β-Blocker Cholesterol-Lowering Asymptomatic Plaque Study (BCAPS). 2001;103:1721-6. doi: 10.1161/01.cir.103.13.1721.

15. Ruggenenti P, Fassi A, Ilieva AP, Bruno S, Iliev IP, Brusegan V, et al. Preventing microalbuminuria in type 2 diabetes. N Engl J Med. 2004;351:1941-51. doi: 10.1056/NEJMoa042167.

16. Ruggenenti P, Fassi A, Ilieva A, Iliev IP, Chiurchiu C, Rubis N, et al. Effects of verapamil added-on trandolapril therapy in hypertensive type 2 diabetes patients with microalbuminuria: the BENEDICT-B randomized trial. J Hypertens. 2011;29:207-16.

17. β-Blocker Heart Attack Trial Research Group. A randomized trial of propranolol in patients with acute myocardial infarction. II. Morbidity results. JAMA. 1983;250:2814-9.

18. β-Blocker Heart Attack Trial Research Group. A randomized trial of propranolol in patients with acute myocardial infarction. I. Mortality results. JAMA. 1982;247:1707-14.

19. Nissen SE, Tuzcu E, Libby P, et al. Effect of antihypertensive agents on cardiovascular events in patients with coronary disease and normal blood pressure: The camelot study: a randomized controlled trial. JAMA. 2004;292:2217-25. doi: 10.1001/jama.292.18.2217.

20. Verdecchia P, Staessen JA, Angeli F, de Simone G, Achilli A, Ganau A, et al. Usual versus tight control of systolic blood pressure in non-diabetic patients with hypertension (Cardio-Sis): an open-label randomised trial. Lancet (London, England). 2009;374:525-33. doi: 10.1016/s0140-6736(09)61340-4.

21. The Danish Study Group on Verapamil in Myocardial Infarction. Effect of verapamil on mortality and major events after acute myocardial infarction (The Danish Verapamil Infarction Trial II — DAVIT II). The American Journal of Cardiology. 1990;66:779-85. doi: 10.1016/0002-9149(90)90351-Z.

22. Ruggenenti P, Lauria G, Iliev IP, Fassi A, Ilieva AP, Rota S, et al. Effects of manidipine and delapril in hypertensive patients with type 2 diabetes mellitus: the delapril and manidipine for nephroprotection in diabetes (DEMAND) randomized clinical trial. Hypertension. 2011;58:776-83. doi: 10.1161/hypertensionaha.111.174474.

23. Marre M, Lievre M, Chatellier G, Mann JF, Passa P, Menard J, et al. Effects of low dose ramipril on cardiovascular and renal outcomes in patients with type 2 diabetes and raised excretion of urinary albumin: randomised, double blind, placebo controlled trial (the DIABHYCAR study). BMJ. 2004;328:495. doi: 10.1136/bmj.37970.629537.0D.

24. Chaturvedi N, Porta M, Klein R, Orchard T, Fuller J, Parving HH, et al. Effect of candesartan on prevention (DIRECT-Prevent 1) and progression (DIRECT-Protect 1) of retinopathy in type 1 diabetes: randomised, placebo-controlled trials. The Lancet. 2008;372:1394-402. doi: 10.1016/s0140-6736(08)61412-9.

25. Sjølie AK, Klein R, Porta M, Orchard T, Fuller J, Parving HH, et al. Effect of candesartan on progression and regression of retinopathy in type 2 diabetes (DIRECT-Protect 2): a randomised placebo-controlled trial. The Lancet. 2008;372:1385-93. doi: 10.1016/s0140-6736(08)61411-7.

26. Tillin T, Orchard T, Malm A, Fuller J, Chaturvedi N. The role of antihypertensive therapy in reducing vascular complications of type 2 diabetes. Findings from the DIabetic REtinopathy Candesartan Trials-Protect 2 study. Journal of hypertension. 2011;29:1457-62. doi: 10.1097/HJH.0b013e3283480db9.

27. Bosch J, Yusuf S, Gerstein HC, Pogue J, Sheridan P, Dagenais G, et al. Effect of ramipril on the incidence of diabetes. N Engl J Med. 2006;355:1551-62. doi: 10.1056/NEJMoa065061.

28. The Dutch TIA Trial Study Group. Trial of secondary prevention with atenolol after transient ischemic attack or nondisabling ischemic stroke. Stroke. 1993;24:543-8. doi: 10.1161/01.str.24.4.543.

29. The EURopean trial On reduction of cardiac events with Perindopril in stable coronary Artery disease Investigators. Efficacy of perindopril in reduction of cardiovascular events among patients with stable coronary artery disease: randomised, double-blind, placebo-controlled, multicentre trial (the EUROPA study). The Lancet. 2003;362:782-8. doi: 10.1016/S0140-6736(03)14286-9.

30. Amery A, Birkenhager W, Brixko P, Bulpitt C, Clement D, Deruyttere M, et al. Mortality and morbidity results from the European Working Party on High Blood Pressure in the Elderly trial. Lancet. 1985;1:1349-54.

31. Liu L, Zhang Y, Liu G, Li W, Zhang X, Zanchetti A. The Felodipine Event Reduction (FEVER) Study: a randomized long-term placebo-controlled trial in Chinese hypertensive patients. J Hypertens. 2005;23:2157-72.

32. Fogari R, Preti P, Zoppi A, Rinaldi A, Corradi L, Pasotti C, et al. Effects of amlodipine fosinopril combination on microalbuminuria in hypertensive type 2 diabetic patients. Am J Hypertens. 2002;15:1042-9.

33. Hypertension Detection and Follow-up Program Cooperative Group. Five-year findings of the hypertension detection and follow-up program. I. Reduction in mortality of persons with high blood pressure, including mild hypertension. JAMA. 1979;277:157-66.

34. Coope J, Warrender TS. Randomised trial of treatment of hypertension in elderly patients in primary care. Br Med J (Clin Res Ed). 1986;293:1145-51. doi: 10.1136/bmj.293.6555.1145.

35. Asayama K, Ohkubo T, Metoki H, Obara T, Inoue R, Kikuya M, et al. Cardiovascular outcomes in the first trial of antihypertensive therapy guided by self-measured home blood pressure. Hypertens Res. 2012;35:1102-10. doi: 10.1038/hr.2012.125.

36. Yusuf S, Sleight P, Pogue J, Bosch J, Davies R, Dagenais G. Effects of an angiotensin-converting-enzyme inhibitor, ramipril, on cardiovascular events in high-risk patients. N Engl J Med. 2000;342:145-53. doi: 10.1056/nejm200001203420301.

37. Lonn EM, Bosch J, López-Jaramillo P, Zhu J, Liu L, Pais P, et al. Blood-Pressure Lowering in Intermediate-Risk Persons without Cardiovascular Disease. N Engl J Med. 2016;374:2009-20. doi: doi:10.1056/NEJMoa1600175.

38. Hansson L, Zanchetti A, Carruthers SG, Dahlöf B, Elmfeldt D, Julius S, et al. Effects of intensive blood-pressure lowering and low-dose aspirin in patients with hypertension: principal results of the Hypertension Optimal Treatment (HOT) randomised trial. The Lancet. 1998;351:1755-62. doi: 10.1016/s0140-6736(98)04311-6.

39. Hypertension-Stroke Cooperative Study Group. Effect of antihypertensive treatment on stroke recurrence. JAMA. 1974;229:409-18.

40. Sun M, Zhou H, Jia Z. Prevention and treatment of stroke after hypertension for ten years in Hunan Province. Zhonghua Nei Ke Za Zhi. 1997;36:312-4.

41. Beckett NS, Peters R, Fletcher AE, Staessen JA, Liu L, Dumitrascu D, et al. Treatment of hypertension in patients 80 years of age or older. N Engl J Med. 2008;358:1887-98. doi: 10.1056/NEJMoa0801369.

42. Bulpitt CJ, Beckett NS, Cooke J, Dumitrascu DL, Gil-Extremera B, Nachev C, et al. Results of the pilot study for the Hypertension in the Very Elderly Trial. J Hypertens. 2003;21:2409-17. doi: 10.1097/01.hjh.0000084782.15238.a2.

43. Lewis EJ, Hunsicker LG, Clarke WR, Berl T, Pohl MA, Lewis JB, et al. Renoprotective Effect of the Angiotensin-Receptor Antagonist Irbesartan in Patients with Nephropathy Due to Type 2 Diabetes. N Engl J Med. 2001;345:851-60. doi: 10.1056/NEJMoa011303.

44. Berl T, Hunsicker LG, Lewis JB, et al. CArdiovascular outcomes in the irbesartan diabetic nephropathy trial of patients with type 2 diabetes and overt nephropathy. Ann Intern Med. 2003;138:542-9. doi: 10.7326/0003-4819-138-7-200304010-00010.

45. Rouleau JL, Warnica WJ, Baillot R, Block PJ, Chocron S, Johnstone D, et al. Effects of angiotensin-converting enzyme inhibition in low-risk patients early after coronary artery bypass surgery. Circulation. 2008;117:24-31. doi: 10.1161/circulationaha.106.685073.

46. The IPPPSH Collaborative Group. Cardiovascular risk and risk factors in a randomized trial of treatment based on the beta-blocker oxprenolol: the International Prospective Primary Prevention Study in Hypertension (IPPPSH). J Hypertens. 1985;3:379-92.

47. Parving HH, Lehnert H, Brochner-Mortensen J, Gomis R, Andersen S, Arner P. The effect of irbesartan on the development of diabetic nephropathy in patients with type 2 diabetes. N Engl J Med. 2001;345:870-8. doi: 10.1056/NEJMoa011489.

48. Jatos Study Group. Principal Results of the Japanese Trial to Assess Optimal Systolic Blood Pressure in Elderly Hypertensive Patients (JATOS). Hypertens Res. 2008;31:2115. doi: 10.1291/hypres.31.2115.

49. Lewis EJ, Hunsicker LG, Bain RP, Rohde RD. The effect of angiotensin-converting-enzyme inhibition on diabetic nephropathy. The Collaborative Study Group. N Engl J Med. 1993;329:1456-62. doi: 10.1056/nejm199311113292004.

50. The MACB Study Group. Effect of metoprolol on death and cardiac events during a 2-year period after coronary artery bypass grafting. Eur Heart J. 1995;16:1825-32.

51. Medical Research Council Working Party. MRC trial of treatment of mild hypertension: principal results. Br Med J (Clin Res Ed). 1985;291:97-104.

52. MRC Working Party. Medical Research Council trial of treatment of hypertension in older adults: principal results BMJ. 1992;304:405-12.

53. A multicentre international study. Improvement in prognosis of myocardial infarction by long-term beta-adrenoreceptor blockade using practolol. Br Med J. 1975;3:735-40.

54. McMurray JJ, Holman RR, Haffner SM, Bethel MA, Holzhauer B, Hua TA, et al. Effect of valsartan on the incidence of diabetes and cardiovascular events. N Engl J Med. 2010;362:1477-90. doi: 10.1056/NEJMoa1001121.

55. The ONTARGET Investigators. Telmisartan, Ramipril, or Both in Patients at High Risk for Vascular Events. N Engl J Med. 2008;358:1547-59. doi: 10.1056/NEJMoa0801317.

56. Imai E, Chan JC, Ito S, Yamasaki T, Kobayashi F, Haneda M, et al. Effects of olmesartan on renal and cardiovascular outcomes in type 2 diabetes with overt nephropathy: a multicentre, randomised, placebo-controlled study. Diabetologia. 2011;54:2978-86. doi: 10.1007/s00125-011-2325-z.

57. Ogawa H, Kim-Mitsuyama S, Matsui K, Jinnouchi T, Jinnouchi H, Arakawa K, et al. Angiotensin II receptor blocker-based therapy in Japanese elderly, high-risk, hypertensive patients. Am J Med. 2012;125:981-90. doi: 10.1016/j.amjmed.2011.12.010.

58. Helgeland A. Treatment of mild hypertension: a five year controlled drug trial. The Oslo study. Am J Med. 1980;69:725-32.

59. MacMahon S, Sharpe N, Gamble G, Clague A, Mhurchu CN, Clark T, et al. Randomized, placebo-controlled trial of the angiotensin-converting enzyme inhibitor, ramipril, in patients with coronary or other occlusive arterial disease. PART-2 Collaborative Research Group. Prevention of Atherosclerosis with Ramipril. J Am Coll Cardiol. 2000;36:438-43.

60. Liu L, Wang Z, Gong L, Zhang Y, Thijs L, Staessen JA, et al. Blood pressure reduction for the secondary prevention of stroke: a Chinese trial and a systematic review of the literature. Hypertension research : official journal of the Japanese Society of Hypertension. 2009;32:1032-40. doi: 10.1038/hr.2009.139.

61. Braunwald E, Domanski MJ, Fowler SE, Geller NL, Gersh BJ, Hsia J, et al. Angiotensin-converting-enzyme inhibition in stable coronary artery disease. N Engl J Med. 2004;351:2058-68. doi: 10.1056/NEJMoa042739.

62. Luders S, Schrader J, Berger J, Unger T, Zidek W, Bohm M, et al. The PHARAO study: prevention of hypertension with the angiotensin-converting enzyme inhibitor ramipril in patients with high-normal blood pressure: a prospective, randomized, controlled prevention trial of the German Hypertension League. J Hypertens. 2008;26:1487-96. doi: 10.1097/HJH.0b013e3282ff8864.

63. Asselbergs FW, Diercks GF, Hillege HL, van Boven AJ, Janssen WM, Voors AA, et al. Effects of fosinopril and pravastatin on cardiovascular events in subjects with microalbuminuria. Circulation. 2004;110:2809-16. doi: 10.1161/01.CIR.0000146378.65439.7A.

64. Pitt B, Byington RP, Furberg CD, Hunninghake DB, Mancini GB, Miller ME, et al. Effect of amlodipine on the progression of atherosclerosis and the occurrence of clinical events. PREVENT Investigators. Circulation. 2000;102:1503-10.

65. Fuchs SC, Poli-de-Figueiredo CE, Figueiredo Neto JA, Scala LC, Whelton PK, Mosele F, et al. Effectiveness of Chlorthalidone Plus Amiloride for the Prevention of Hypertension: The PREVER-Prevention Randomized Clinical Trial. J Am Heart Assoc. 2016;5. doi: 10.1161/jaha.116.004248.

66. Yusuf S, Diener HC, Sacco RL, Cotton D, Ounpuu S, Lawton WA, et al. Telmisartan to prevent recurrent stroke and cardiovascular events. N Engl J Med. 2008;359:1225-37. doi: 10.1056/NEJMoa0804593.

67. PROGRESS Collaborative Group. Randomised trial of a perindopril-based blood-pressure-lowering regimen among 6105 individuals with previous stroke or transient ischaemic attack. The Lancet. 2001;358:1033-41. doi: 10.1016/S0140-6736(01)06178-5.

68. Pitt B, O’Neill B, Feldman R, Ferrari R, Schwartz L, Mudra H, et al. The Quinapril Ischemic Event Trial (QUIET): evaluation of chronic ace inhibitor therapy in patients with ischemic heart disease and preserved left ventricular function. The American Journal of Cardiology. 2001;87:1058-63. doi: 10.1016/S0002-9149(01)01461-8.

69. Mauer M, Zinman B, Gardiner R, Suissa S, Sinaiko A, Strand T, et al. Renal and retinal effects of enalapril and losartan in type 1 diabetes. N Engl J Med. 2009;361:40-51. doi: 10.1056/NEJMoa0808400.

70. Ravid M, Brosh D, Levi Z, Bar-Dayan Y, Ravid D, Rachmani R. Use of enalapril to attenuate decline in renal function in normotensive, normoalbuminuric patients with type 2 diabetes mellitus. A randomized, controlled trial. Ann Intern Med. 1998;128:982-8.

71. Brenner BM, Cooper ME, de Zeeuw D, Keane WF, Mitch WE, Parving HH, et al. Effects of losartan on renal and cardiovascular outcomes in patients with type 2 diabetes and nephropathy. N Engl J Med. 2001;345:861-9. doi: 10.1056/NEJMoa011161.

72. Haller H, Ito S, Izzo JL, Jr., Januszewicz A, Katayama S, Menne J, et al. Olmesartan for the delay or prevention of microalbuminuria in type 2 diabetes. N Engl J Med. 2011;364:907-17. doi: 10.1056/NEJMoa1007994.

73. Teo KK, Burton JR, Buller CE, Plante S, Catellier D, Tymchak W, et al. Long-term effects of cholesterol lowering and angiotensin-converting enzyme inhibition on coronary atherosclerosis: The Simvastatin/Enalapril Coronary Atherosclerosis Trial (SCAT). Circulation. 2000;102:1748-54.

74. Lithell H, Hansson L, Skoog I, Elmfeldt D, Hofman A, Olofsson B, et al. The Study on Cognition and Prognosis in the Elderly (SCOPE): principal results of a randomized double-blind intervention trial. J Hypertens. 2003;21:875-86.

75. SHEP Cooperative Research Group. Prevention of stroke by antihypertensive drug treatment in older persons with isolated systolic hypertension: Final results of the systolic hypertension in the elderly program (shep). JAMA. 1991;265:3255-64. doi: 10.1001/jama.1991.03460240051027.

76. Curb JD, Pressel SL, Cutler JA, Savage PJ, Applegate WB, Black H, et al. Effect of diuretic-based antihypertensive treatment on cardiovascular disease risk in older diabetic patients with isolated systolic hypertension. Systolic Hypertension in the Elderly Program Cooperative Research Group. JAMA. 1996;276:1886-92.

77. Perry HM, Smith WM, McDonald RH, Black D, Cutler JA, Furberg CD, et al. Morbidity and mortality in the Systolic Hypertension in the Elderly Program (SHEP) pilot study. Stroke. 1989;20:4-13. doi: 10.1161/01.str.20.1.4.

78. The SPRINT Research Group. A Randomized Trial of Intensive versus Standard Blood-Pressure Control. N Engl J Med. 2015;373:2103-16. doi: 10.1056/NEJMoa1511939.

79. The SPS3 Study Group. Blood-pressure targets in patients with recent lacunar stroke: the SPS3 randomised trial. The Lancet. 2013;382:507-15. doi: 10.1016/S0140-6736(13)60852-1.

80. Gong L, Zhang W, Zhu Y, Zhu J, Kong D, Page V, et al. Shanghai trial of nifedipine in the elderly (STONE). J Hypertens. 1996;14:1237-45.

81. Dahlöf B, Hansson L, Lindholm LH, Scherstén B, Ekbom T, Wester PO. Morbidity and mortality in the Swedish Trial in Old Patients with Hypertension (STOP-Hypertension). The Lancet. 1991;338:1281-5. doi: 10.1016/0140-6736(91)92589-T.

82. Liu L, Wang JG, Gong L, Liu G, Staessen JA. Comparison of active treatment and placebo in older Chinese patients with isolated systolic hypertension. Systolic Hypertension in China (Syst-China) Collaborative Group. J Hypertens. 1998;16:1823-9.

83. Wang JG, Staessen JA, Gong L, Liu L. Chinese trial on isolated systolic hypertension in the elderly. Systolic Hypertension in China (Syst-China) Collaborative Group. Arch Intern Med. 2000;160:211-20.

84. Staessen JA, Fagard R, Thijs L, Celis H, Arabidze GG, Birkenhager WH, et al. Randomised double-blind comparison of placebo and active treatment for older patients with isolated systolic hypertension. The Systolic Hypertension in Europe (Syst-Eur) Trial Investigators. Lancet. 1997;350:757-64.

85. Eriksson S, Olofsson B, Wester P. Atenolol in Secondary Prevention after Stroke. Cerebrovasc Dis. 1995;5:21-5.

86. The Telmisartan Randomised AssessmeNt Study in ACE iNtolerant subjects with cardiovascular Disease (TRANSCEND) Investigators. Effects of the angiotensin-receptor blocker telmisartan on cardiovascular events in high-risk patients intolerant to angiotensin-converting enzyme inhibitors: a randomised controlled trial. The Lancet. 2008;372:1174-83. doi: 10.1016/S0140-6736(08)61242-8.

87. U. K. Prospective Diabetes Study Group. Tight blood pressure control and risk of macrovascular and microvascular complications in type 2 diabetes: UKPDS 38. BMJ : British Medical Journal. 1998;317:703-13.

88. Fried LF, Emanuele N, Zhang JH, Brophy M, Conner TA, Duckworth W, et al. Combined angiotensin inhibition for the treatment of diabetic nephropathy. N Engl J Med. 2013;369:1892-903. doi: 10.1056/NEJMoa1303154.

89. Veterans Administration Cooperative Study Group on Antihypertensive Agents. Effects of treatment on morbidity in hypertension. II. Results in patients with diastolic blood pressure averaging 90 through 114 mm Hg. JAMA. 1970;213:1143-52.

90. Ogihara T, Saruta T, Rakugi H, Matsuoka H, Shimamoto K, Shimada K, et al. Target blood pressure for treatment of isolated systolic hypertension in the elderly: valsartan in elderly isolated systolic hypertension study. Hypertension. 2010;56:196-202. doi: 10.1161/HYPERTENSIONAHA.109.146035.

91. Wei Y, Jin Z, Shen G, Zhao X, Yang W, Zhong Y, et al. Effects of intensive antihypertensive treatment on Chinese hypertensive patients older than 70 years. J Clin Hypertens (Greenwich). 2013;15:420-7. doi: 10.1111/jch.12094.
