# Supplementary material for: Are the effects of blood pressure lowering treatment diminishing?: meta-regression analyses
Source: Clin Hypertens. 2018 Nov 15;24:16. doi: 10.1186/s40885-018-0101-9 (PMC6237040; doi:10.1186/s40885-018-0101-9)
Supplement: Supplementary file 2 — Table S2. Summary of Outcome Events on All-cause Mortality. (DOCX 28 kb) [file 40885_2018_101_MOESM2_ESM.docx]

**Additional file 2**

Table S2. Summary of Outcome Events on All-cause Mortality

| Trial Name | Events Intervention | Participants Intervention | Events Control | Participants Control |
| --- | --- | --- | --- | --- |
| AASK | 44 | 540 | 61 | 554 |
| ABCD-H | 13 | 237 | 25 | 233 |
| ABCD-N | 18 | 237 | 20 | 243 |
| ACCORD | 150 | 2362 | 144 | 2371 |
| ACTION | 310 | 3825 | 291 | 3840 |
| ACTIVE I | 949 | 4518 | 929 | 4498 |
| ADVANCE | 408 | 5569 | 471 | 5571 |
| AIPRI | 8 | 300 | 1 | 283 |
| ALTITUDE | 376 | 4274 | 358 | 4287 |
| ANBPS | 25 | 1721 | 35 | 1706 |
| BCAPS | 4 | 396 | 7 | 397 |
| BENEDICT | 7 | 904 | 5 | 300 |
| BENEDICT-B | 2 | 138 | 7 | 143 |
| BHAT | 138 | 1916 | 188 | 1921 |
| CAMELOT | 15 | 1336 | 6 | 655 |
| Cardio-Sis | 4 | 558 | 5 | 553 |
| DAVIT 2 | 95 | 878 | 119 | 897 |
| DEMAND | 3 | 253 | 3 | 127 |
| DIABHYCAR | 334 | 2443 | 324 | 2469 |
| DIRECT PREVENT 1 | 7 | 711 | 5 | 710 |
| DIRECT-PROTECT 1 | 7 | 951 | 8 | 954 |
| DIRECT-PROTECT 2 | 37 | 951 | 35 | 954 |
| DREAM | 31 | 2623 | 32 | 2646 |
| Dutch TIA | 64 | 732 | 58 | 741 |
| EUROPA | 375 | 6110 | 420 | 6108 |
| EWPHE | 135 | 416 | 149 | 424 |
| FEVER | 112 | 4841 | 151 | 4870 |
| Fogari-02 | 2 | 104 | 7 | 205 |
| HDFP | 349 | 5485 | 419 | 5455 |
| HEP | 60 | 419 | 69 | 465 |
| HOMED-BP | 27 | 1759 | 31 | 1759 |
| HOPE | 482 | 4645 | 569 | 4652 |
| HOPE-3 | 342 | 6356 | 349 | 6349 |
| HOT | 401 | 12526 | 188 | 6264 |
| HSCS | 26 | 233 | 24 | 219 |
| HYVET | 196 | 1933 | 235 | 1912 |
| HYVET pilot | 57 | 857 | 22 | 426 |
| IDNT | 170 | 1146 | 93 | 569 |
| IMAGINE | 28 | 1280 | 28 | 1273 |
| IPPPSH | 108 | 3185 | 114 | 3172 |
| IRMA-2 | 3 | 389 | 1 | 201 |
| JATOS | 54 | 2212 | 42 | 2206 |
| Lewis-93 | 8 | 207 | 14 | 202 |
| MACB | 23 | 480 | 13 | 487 |
| MRC-1 | 248 | 8700 | 253 | 8654 |
| MRC-2 | 301 | 2183 | 315 | 2213 |
| MultiCentre Int | 52 | 1524 | 76 | 1514 |
| NAVIGATOR | 295 | 4631 | 327 | 4675 |
| ONTARGET | 1065 | 8502 | 2003 | 17118 |
| ORIENT | 19 | 282 | 20 | 284 |
| Oslo | 10 | 406 | 9 | 379 |
| PART-2 | 16 | 308 | 25 | 309 |
| PATS | 145 | 2840 | 161 | 2825 |
| PEACE | 299 | 4158 | 334 | 4132 |
| PHARAO | 5 | 505 | 2 | 503 |
| PREVEND IT | 13 | 431 | 12 | 433 |
| PREVENT | 6 | 417 | 8 | 408 |
| PREVER | 1 | 372 | 1 | 358 |
| PRoFESS | 755 | 10146 | 740 | 10186 |
| PROGRESS | 306 | 3051 | 319 | 3054 |
| QUIET | 27 | 878 | 27 | 872 |
| RASS | 2 | 190 | 1 | 95 |
| RAVID-98 | 3 | 97 | 2 | 97 |
| RENAAL | 158 | 751 | 155 | 762 |
| ROADMAP | 26 | 2232 | 15 | 2215 |
| SCAT | 8 | 229 | 11 | 231 |
| SCOPE | 259 | 2477 | 266 | 2460 |
| SHEP | 213 | 2365 | 242 | 2371 |
| SHEP pilot | 32 | 443 | 7 | 108 |
| SPRINT | 155 | 4678 | 210 | 4683 |
| SPS3 | 106 | 1501 | 101 | 1519 |
| STONE | 15 | 891 | 26 | 741 |
| STOP | 36 | 812 | 63 | 815 |
| Syst-China | 61 | 1253 | 82 | 1141 |
| Syst-Eur | 123 | 2398 | 137 | 2297 |
| TEST | 51 | 372 | 60 | 348 |
| TRANSCEND | 364 | 2954 | 349 | 2972 |
| UKPDS 38 | 134 | 758 | 83 | 390 |
| VA NEPHRON-D | 63 | 724 | 60 | 724 |
| VA-2 | 10 | 186 | 21 | 194 |
| VALISH | 24 | 1545 | 30 | 1534 |
| Wei-13 | 51 | 363 | 87 | 361 |
