# Supplementary material for: Are the effects of blood pressure lowering treatment diminishing?: meta-regression analyses
Source: Clin Hypertens. 2018 Nov 15;24:16. doi: 10.1186/s40885-018-0101-9 (PMC6237040; doi:10.1186/s40885-018-0101-9)
Supplement: Supplementary file 3 — Table S3. Summary of Outcome Events on Stroke. (DOCX 28 kb) [file 40885_2018_101_MOESM3_ESM.docx]

**Additional file 3**

Table S3. Summary of Outcome Events on Stroke

| Trial Name | Events Intervention | Participants Intervention | Events Control | Participants Control |
| --- | --- | --- | --- | --- |
| AASK | 26 | 540 | 29 | 554 |
| ABCD-H | 9 | 237 | 9 | 233 |
| ABCD-N | 4 | 237 | 13 | 243 |
| ACCORD | 36 | 2362 | 62 | 2371 |
| ACTION | 82 | 3825 | 108 | 3840 |
| ACTIVE I | 379 | 4518 | 411 | 4498 |
| ADVANCE | 215 | 5569 | 218 | 5571 |
| AIPRI | 2 | 300 | 3 | 283 |
| ALTITUDE | 147 | 4274 | 122 | 4287 |
| ANBPS | 13 | 1721 | 22 | 1706 |
| BBB | 8 | 1064 | 11 | 1064 |
| BCAPS | 1 | 396 | 7 | 397 |
| BENEDICT-B | 5 | 138 | 4 | 143 |
| BHAT | 29 | 1916 | 30 | 1921 |
| CAMELOT | 14 | 1336 | 12 | 655 |
| Cardio-Sis | 4 | 558 | 9 | 553 |
| DIABHYCAR | 118 | 2443 | 116 | 2469 |
| DIRECT-PROTECT 2 | 16 | 951 | 15 | 954 |
| DREAM | 4 | 2623 | 8 | 2646 |
| Dutch TIA | 52 | 732 | 62 | 741 |
| EUROPA | 98 | 6110 | 102 | 6108 |
| EWPHE | 21 | 416 | 31 | 424 |
| FEVER | 177 | 4841 | 251 | 4870 |
| Fogari-02 | 1 | 104 | 5 | 205 |
| HDFP | 29 | 5485 | 52 | 5455 |
| HEP | 23 | 419 | 44 | 465 |
| HOMED-BP | 20 | 1759 | 16 | 1759 |
| HOPE | 156 | 4645 | 226 | 4652 |
| HOPE-3 | 75 | 6356 | 94 | 6349 |
| HOT | 200 | 12526 | 94 | 6264 |
| HSCS | 37 | 233 | 42 | 219 |
| Hunan Province | 37 | 1040 | 79 | 1040 |
| HYVET | 51 | 1933 | 69 | 1912 |
| HYVET pilot | 18 | 857 | 18 | 426 |
| IDNT | 43 | 1146 | 26 | 569 |
| IMAGINE | 15 | 1280 | 14 | 1273 |
| IPPPSH | 45 | 3185 | 46 | 3172 |
| JATOS | 44 | 2212 | 42 | 2206 |
| MACB | 3 | 480 | 3 | 487 |
| MRC-1 | 60 | 8700 | 109 | 8654 |
| MRC-2 | 101 | 2183 | 134 | 2213 |
| NAVIGATOR | 105 | 4631 | 132 | 4675 |
| ONTARGET | 373 | 8502 | 774 | 17118 |
| ORIENT | 8 | 282 | 11 | 284 |
| OSCAR | 15 | 586 | 24 | 578 |
| Oslo | 0 | 406 | 5 | 379 |
| PART-2 | 7 | 308 | 4 | 309 |
| PATS | 159 | 2840 | 219 | 2825 |
| PEACE | 71 | 4158 | 92 | 4132 |
| PHARAO | 3 | 505 | 1 | 503 |
| PREVEND IT | 1 | 431 | 10 | 433 |
| PREVENT | 5 | 417 | 5 | 408 |
| PRoFESS | 880 | 10146 | 934 | 10186 |
| PROGRESS | 307 | 3051 | 420 | 3054 |
| QUIET | 1 | 878 | 1 | 872 |
| RASS | 1 | 190 | 0 | 95 |
| ROADMAP | 2 | 2232 | 2 | 2215 |
| SCAT | 2 | 229 | 9 | 231 |
| SCOPE | 89 | 2477 | 115 | 2460 |
| SHEP | 103 | 2365 | 159 | 2371 |
| SHEP pilot | 11 | 443 | 6 | 108 |
| SPRINT | 62 | 4678 | 70 | 4683 |
| SPS3 | 125 | 1501 | 152 | 1519 |
| STONE | 16 | 891 | 36 | 741 |
| STOP | 29 | 812 | 53 | 815 |
| Syst-China | 45 | 1253 | 59 | 1141 |
| Syst-Eur | 47 | 2398 | 77 | 2297 |
| TEST | 81 | 372 | 75 | 348 |
| TRANSCEND | 112 | 2954 | 136 | 2972 |
| UKPDS 38 | 38 | 758 | 34 | 390 |
| VA NEPHRON-D | 18 | 724 | 18 | 724 |
| VA-2 | 5 | 186 | 20 | 194 |
| VALISH | 16 | 1545 | 23 | 1534 |
| Wei-13 | 21 | 363 | 36 | 361 |
